# Diseased Teeth and Diseases

**Published:** 1919-03

**Authors:** 


### Diseased Teeth and Diseases.

It is now pretty generally known among the profession and is coming to be known among the laity that diseased teeth are responsible for many of the diseases of the people. People who have diseased mouths cannot stay well. The teeth are the most common causes of infection, and utmost care should be taken to see that they are kept clean and sound. If they are properly cleaned from early childhood dozens of the little and big ills to which "flesh is heir" will be avoided. Many of the tooth washes and nostrums advertised are more harmful than beneficial. It pays to know what you are using in the way of a dentifrice, to get the best and to take no substitutes. You cannot afford to be careless as to the care of the teeth through which much infection can be carried into the body. Believing that an ounce of prevention is worth a pound of cure, the Texas Medical Journal is going to conduct a campaign of several months to induce the people to properly care for their teeth. The way to reach them is through the physicians and dentists of the country. Something will be said on this subject every month until the people who are influenced to any extent directly or indirectly by the Texas Medical Journal will come to a proper appreciation of the care of the teeth.

